# Electrochemical deposition of mesoporous high-entropy Pt–Pd–Rh–Ru–Cu–Au–Se–Mo films using polymeric micelle templating

**DOI:** 10.1039/d5sc04126k

**Published:** 2025-09-23

**Authors:** Yoto Saso, Yunqing Kang, Lei Fu, Kotaro Yagi, Jungmok You, Yusuke Asakura, Yusuke Yamauchi

**Affiliations:** a Department of Materials Process Engineering, Graduate School of Engineering, Nagoya University Nagoya 464−8603 Japan yqkang@toki.waseda.jp; b Department of Convergent Biotechnology and Advanced Materials Science, Kyung Hee University 1732 Deogyeong-daero, Giheung-gu, Yongin-si Gyeonggi-do 17104 South Korea; c Australian Institute for Bioengineering and Nanotechnology (AIBN), The University of Queensland Brisbane Queensland 4072 Australia y.yamauchi@uq.edu.au

## Abstract

Platinum (Pt)-based high-entropy alloys (HEAs) can optimize catalytic properties through multi-element synergy, yet conventional synthesis requires harsh conditions such as high temperature/pressure or toxic solvents, limiting sustainable applications. Here, we report the synthesis of a mesoporous high-entropy alloy composed of Pt, Pd, Rh, Ru, Cu, Au, Se, and Mo using a micelle soft-templating method by electrochemical deposition. The effects of electrodeposition potentials on entropy changes and element content are explored. The typical eight-element HEA exhibits the highest entropy value (>1.95*R*) among reported mesoporous HEAs to date. The mesoporous architecture enhances active sites and mass transport, offering an eco-friendly platform for advanced catalytic materials in energy conversion and storage.

## Abstract 2

A mesoporous high-entropy alloy film (Pt, Pd, Rh, Ru, Cu, Au, Se, Mo) is synthesized *via* soft-templated electrochemical deposition at room temperature, achieving the highest entropy value reported for mesoporous metals to date.

## Graphical abstract

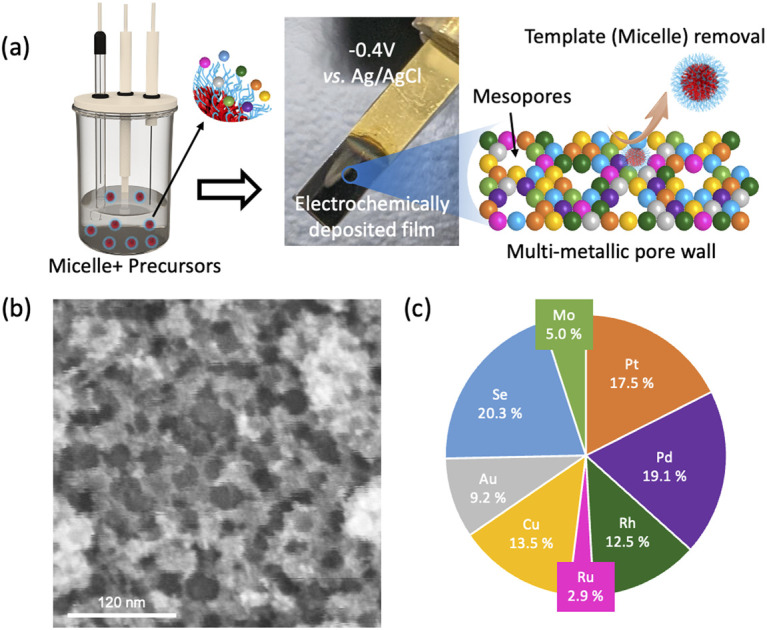

## Introduction

Bottom-up chemical synthesis approaches have been widely employed to design nanoarchitectured metallic materials with potential applications across diverse fields, including catalysis, sensing, and energy storage and conversion.^1^ Platinum (Pt) has long been recognized as a premier catalyst due to its exceptional performance. However, its scarcity and high cost present significant challenges.^2^ To address these issues, the development of nanometallic materials that incorporate Pt with other elements offers a promising strategy to reduce Pt usage while significantly enhancing material performance.^3^ In particular, alloying with additional elements can optimize Pt's electronic structure, leading to improved electrocatalytic performance.^4^

Recently, high-entropy alloys (HEAs) have attracted significant attention for their ability to facilitate complex reactions and enhance catalytic performance.^5–11^ Alloys with a configurational entropy exceeding 1.5*R* (where *R* is the gas constant) are classified as HEAs.^12^ This high configurational entropy, resulting from the incorporation of multiple metal elements, provides a diverse range of active sites that are particularly effective for complex multielectron reactions.^13^ Nanostructure engineering has emerged as a crucial strategy for improving the catalytic properties of HEAs. Many types of nanostructures, including nanoparticles,^14^ nanowires,^15^ and irregular particles,^16^ have been extensively reported to date. However, conventional synthesis methods typically require harsh conditions, such as high temperatures and/or the use of considerable amounts of environmentally harmful organic solvents.^17,18^

To address these challenges, we have developed bottom-up chemical methods for synthesizing mesoporous HEAs (m-HEAs).^19–21^ For example, the electromental deposition method can be conducted at room temperature, involving the electrochemical reduction of metal ions in the presence of polymeric micelles. The key advantage of this approach is that it eliminates the need for the harsh conditions mentioned above, relying instead on a mild, wet-chemical, and soft-template-based synthesis process.^19^ This innovation highlights the critical role of environmentally friendly and scalable techniques in advancing the synthesis of next-generation HEA-based materials. In addition, mesoporous architectures provide a substantially increased electrochemically active surface area, a feature particularly critical for catalytic applications.^22,23^ The interconnected mesopores not only increase the number of active sites available for reactions but also facilitate the efficient diffusion of reactants and products through the porous network.^24^ This combination of high surface area and improved mass transport properties makes mesoporous structures highly advantageous for a wide range of electrochemical applications.^25^

The aim of this study is to synthesize m-HEAs with a high entropy value. We present a robust platform for designing m-HEA films composed of Pt, Pd, Rh, Ru, Cu, Au, Se, and Mo. By carefully controlling the applied potentials, the mixed configuration entropy value (Δ*S*_mix_) exceeds 1.95*R*, which is, to the best of our knowledge, the highest reported for mesoporous materials (Table S1).^19–21,26–33^ Cu, Se, and Mo are selected to be combined with noble metals for their complementary roles in adjusting electronic structure and/or lattice strain, as well as enhancing catalytic activity, enabling cost-effective and durable performance.^34–36^

## Results and discussion

**Fig. 1:**
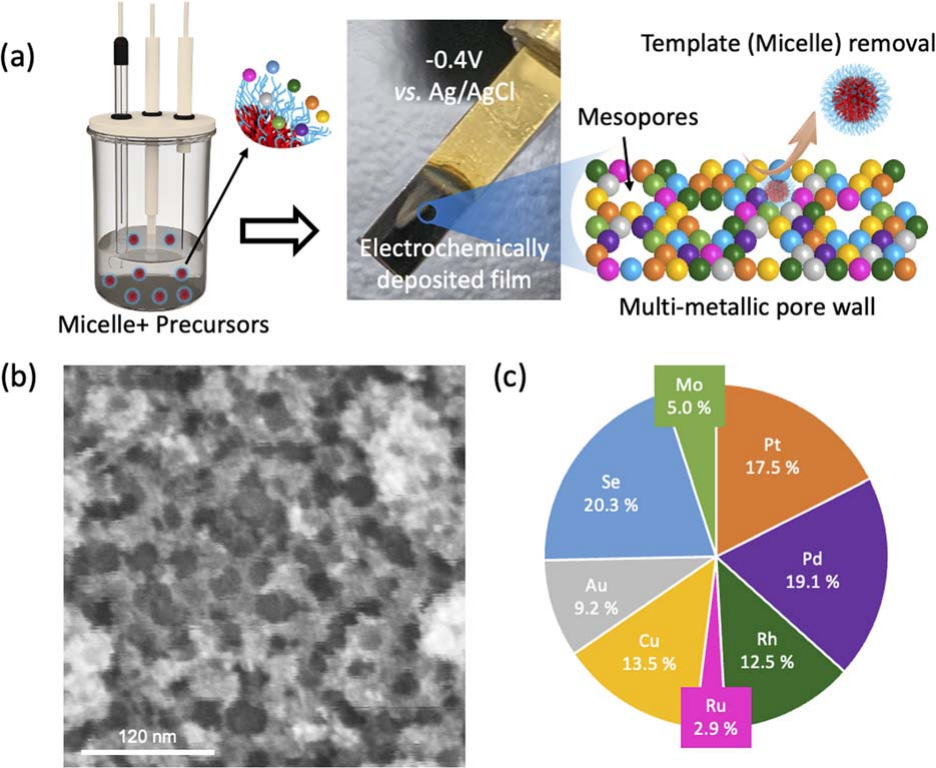
(a) Fabrication of m-HEA thin films using polymeric micelle templates and their structural illustration. (b) Top-view SEM image and (c) elemental molar composition chart of the m-HEA film.

The synthesis of m-HEA is based following steps. First, the PS-*b*-PEO polymer (1.63 × 10^−7^ mol, 4 mg) was dissolved in tetrahydrofuran (THF, 0.6 mL), followed by the dropwise addition of specific amounts of ethanol and water. Subsequently, 2 M hydrochloric acid (HCl) solution (0.08 mL) was added to the mixture. Metal salt solutions were then introduced to prepare the electrolyte for electrodeposition. The metal salt solution contained equimolar amounts of Pt, Pd, Rh, Ru, Cu, Au, Se, and Mo salts, each at a concentration of 40 mM. This approach utilizes an electromotive-force-driven micelle assembly process, where metal deposition and micelle assembly occur concurrently. The poly(styrene)-*b*-poly(etheylene oxide) (PS-*b*-PEO) diblock copolymer was initially dissolved in THF (a good solvent for PS and PEO blocks) as unimers. Upon gradual addition of ethanol followed by aqueous metal solutions, spherical micelles formed due to the decreased solubility of PS cores in water. The hydrophilic PEO coronas interacted with aqua-metal complexes at the micelle periphery. Under applied potential, HEA-loaded composite micelles deposited on the working electrode, and the PS-*b*-PEO template was subsequently removed by THF immersion, leaving behind nanoporous architectures. Then, the prepared electrolyte was used for electrodeposition by applying a fixed voltage (−0.4 V *vs.* Ag/AgCl) for 20 minutes. During this process, Au-Ti-coated silicon substate and carbon paper were served as the working electrode. Before use, the carbon papers were pre-treated by soaking in ethanol for at least 10 minutes and subsequently dried in an oven. After electrodeposition, the resulting substrate loaded sample was immersed in THF to remove the polymer template (Fig. 1a). Unless noted otherwise, the typical sample was prepared under the above conditions (see the Experimental Section for details in the SI).

Enlarged scanning electron microscopy (SEM) observation of the m-HEA deposited at −0.4 V (*vs.* Ag/AgCl) reveals that the surface is not uniformly flat (Fig. 1b). Mesopores are distributed throughout the surface, providing a high exposed surface area. The representative pore diameter and the distance between adjacent pores were calculated by counting 100 mesopores based on the SEM images (Fig. S1). The average pore size is approximately 20.6 nm, which falls within the mesoporous range, while the average distance between adjacent pores is about 26.3 nm (Fig. S1c and d). The cross-section of the m-HEA film was observed in detail using transmission electron microscopy (TEM), revealing a uniformly distributed mesoporous structure throughout the film (Fig. S1b and 2a). The pores exhibit an elliptical shape, distorted perpendicular to the substrate, with the long axis measuring approximately 22.4 nm. This pore diameter closely matches the pore size observed in the above SEM image. In addition, the pore size can be tuned by varying the molecular weight of the PS block in PS-*b*-PEO, with lower molecular weight yielding smaller pores (11.9 nm). In addition, larger pores (32.1 nm) are obtained by introducing a PS swelling agent (Fig. S2).

**Fig. 2:**
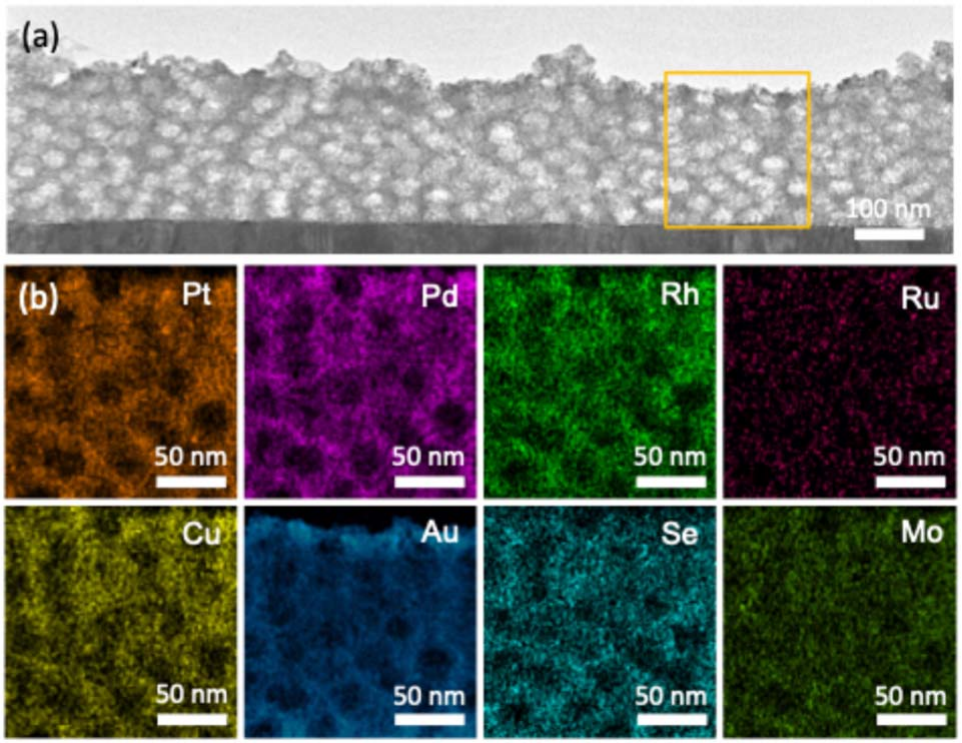
(a) Cross-sectional TEM image and (b) corresponding EDS maps in the selected area in (a) of the m-HEA film.

To investigate mesostructural periodicity, SAXS (small-angle X-ray scattering) measurement was conducted on the m-HEA sample on carbon paper (−0.4 V *vs.* Ag/AgCl). Mesoporous HEA layers are deposited on the surface of carbon fibers without any cracks (Fig. S3a and b). The broad peak center is located around *q* = 0.2 nm^−1^, corresponding to a *d*-spacing of approximately 31.4 nm (Fig. S3c). This suggests that the average distance between mesopores is around 31.4 nm, which aligns with the SEM data (Fig. S3a). Although the pore sizes appear uniform (20.6 nm in diameter) in the SEM images, no distinct peaks are observed in the SAXS measurements. This is because the mesopores, while closely packed, are randomly distributed, leading to non-uniform pore spacing.

Compositional analysis performed using energy-dispersive spectroscopy (EDS) attached to SEM on the top surface of the sample indicates that all elements—Pt, Pd, Rh, Ru, Cu, Au, Se, and Mo—are uniformly dispersed without any evidence of segregation (Fig. 2b). Elemental mapping analysis of the cross-sectional TEM image confirms that each element is homogeneously dispersed across the entire film, indicating that the alloy exhibits a high entropy value. The Au content is slightly concentrated and aggregated on the film surface. This is likely due to the galvanic displacement of Au ions present in the electrolyte by the deposited elements during electrochemical deposition and/or the washing process to remove the templates. While a slight increase in Au concentration is observed near the top film surface interface, no significant compositional variations are detected within the sample interior.

From the inductively coupled plasma (ICP) optical emission spectroscopy results, the overall compositional ratios of Pt, Pd, Rh, Ru, Cu, Au, Se, and Mo are 17.5, 19.1, 12.5, 2.9, 13.5, 9.2, 20.3, and 5.0, respectively, yielding a mixing entropy of 1.95*R* (Table S2). The lower content of Mo and Ru may be due to their more negative redox potentials and the fact that Mo prefers a body-centered cubic (bcc) crystal structure and Ru is hexagonal closed-packed (hcp) one, whereas the other noble metals favor face-centered cubic (fcc) structure. Nevertheless, as we have previously reported, the final elemental composition in complex alloys is governed by a synergy of factors, including redox and applied potentials, interactions between micelles and metal ions, metal surface energies, and intrinsic phase preferences.^19–21^.

The crystalline nature of the samples was characterized by wide-angle X-ray diffraction (XRD). After the m-HEA layer is deposited on carbon paper at a potential of −0.4 V (*vs.* Ag/AgCl), the wide-angle XRD peaks are identical to those of the original carbon paper, with no appearance of new peaks or shifts in peak positions (Fig. S3d). All peaks after metal deposition align precisely with the original pattern of the carbon paper. The incorporation of various elements with differing atomic sizes makes crystallization difficult due to size mismatches. To better resolve the crystal structure, we employed a smoother Au–Ti-coated Si substrate as the conductive substrate. The results show that, although part of the signal is masked by the substrate, the single-phase crystalline structure of the m-HEA film can still be observed, as shown in Fig. S4.

**Fig. 3:**
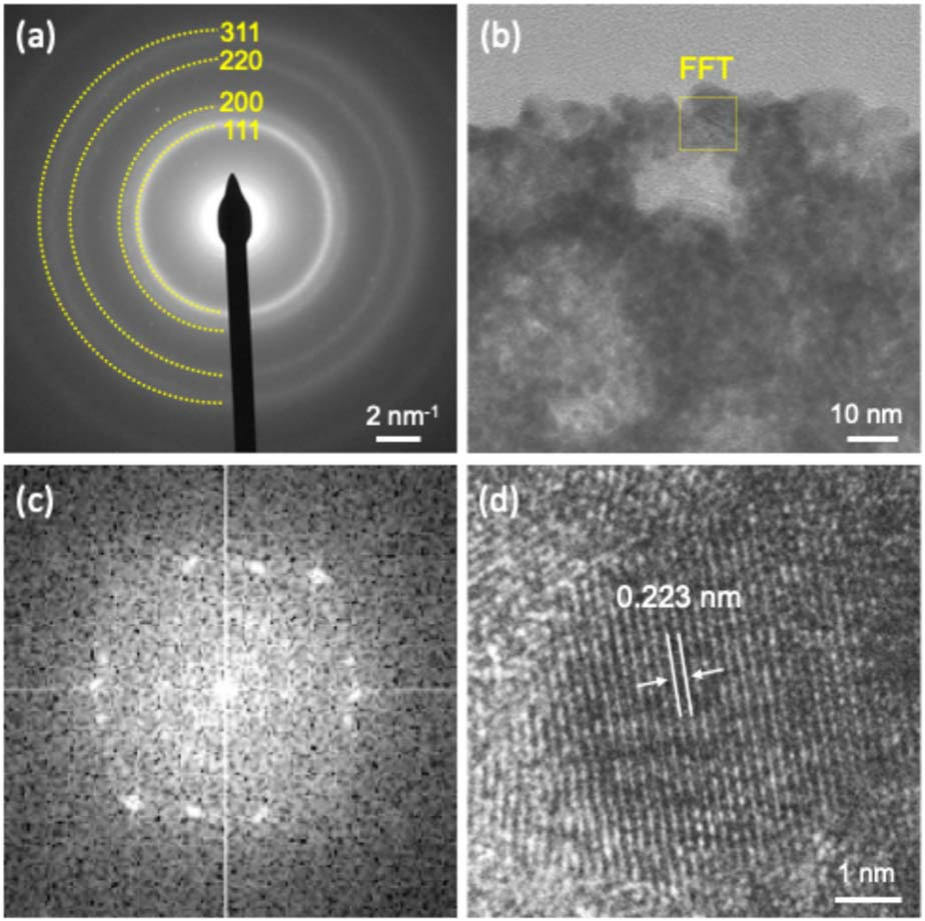
(a) SAED pattern, (b) enlarged TEM image, (c) FFT pattern of the selected area in (b), and (d) HRTEM image of the m-HEA film.

Selected-area electron diffraction (SAED) patterns show ring-like features that can be assigned to an fcc crystal structure of the m-HEA film (Fig. 3a). A high-resolution cross-sectional TEM image clearly reveals that the pore walls are composed of nanoscale crystalline grains (Fig. 3b). Additionally, fast Fourier transform (FFT) analysis shows several spots originating from a polycrystalline structure, indicating that the pore walls consist of extremely small crystalline grains (Fig. 3c). Upon closer observation, as indicated by the square, a lattice spacing of 0.223 nm is distinctly observed (Fig. 3d), which corresponds to the (111) plane at 2*θ* ≈ 40.4° (see the XRD result in Fig. S4).

**Fig. 4:**
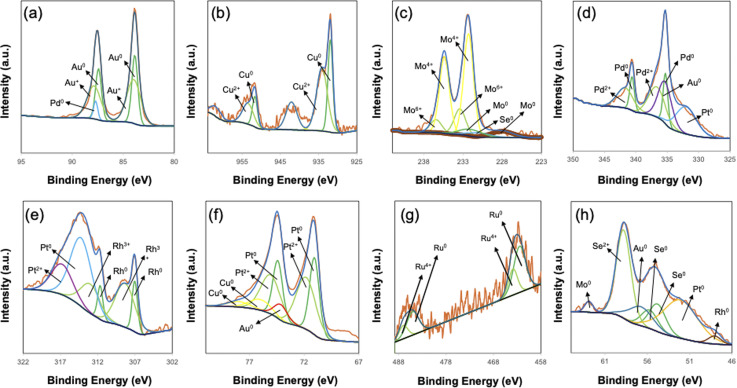
XPS spectra of (a) Au 4f (overlapping with Pd 4s), (b) Cu 2p, (c) Mo 3d (overlapping with Se 3s), (d) Pd 3d (overlapping with Pt 4d and Au 4d), (e) Rh 3d (overlapping with Pt 4d), (f) Pt 4f (overlapping with Cu 3p and Au 5p), (g) Ru 3p, and (h) Se 2p (overlapping with Au 5p, Pt 5p, and Rh 4p) of the m-HEA film.

To confirm the chemical composition and oxidation states of the elements in our m-HEA films, X-ray photoelectron spectroscopy (XPS) was employed. The survey spectrum clearly confirms the presence of Pt, Pd, Rh, Ru, Cu, Au, Se, and Mo within the film (Fig. S5a). High-resolution spectra are subsequently acquired for each element to elucidate their respective chemical states. The high-resolution spectrum of Pt 4f exhibits two peaks corresponding to different spin states. The peak at 71.5 eV corresponds to the metallic (zero-valent) state, while the peak at 72.5 eV corresponds to the divalent oxidized state.^37^ Additionally, overlapping peaks are observed at 74.9 eV for Cu 3p_3/2_, 77.3 eV for Cu 3p_1/2_, and 73.9 eV for Au 5p_3/2_. Similarly, the high-resolution spectrum of Pd 3d contains two peaks, with the peak at 335.3 eV corresponding to the metallic Pd 3d_5/2_ (zero-valent) state and the peak at 338.2 eV corresponding to the divalent oxidized state of Pd. Furthermore, overlapping peaks are observed for Pt 4d_3/2_ and Au 4d_5/2_. The high-resolution spectrum of Rh 3d also exhibits two peaks, with the peak at 307.6 eV corresponding to the metallic Rh 3d_5/2_ (zero-valent) state and the peak at 308.8 eV corresponding to the trivalent oxidized state. Additionally, an overlapping peak for Pt 4d_5/2_ is observed. The spectra of Ru 3p and Cu 2p similarly exhibit two peaks, corresponding to the metallic (zero-valent) and oxidized states. It should be noted that the low-intensity Ru 3p peak is used for fitting due to the overlapping signals of Ru 3d and C 1s (Fig. S5b). The Au 4f spectrum also exhibits two peaks, with Pd 4s overlapping. The Se 3d spectrum contains two peaks corresponding to the metallic (zero-valent) state, while only a single peak is observed for the divalent oxidized state. This indicates that Se has a relatively higher oxidation ratio compared to other metals. The Mo 3d spectrum, like other elements, exhibits two peaks, indicating the presence of Mo in both hexavalent (+6) and tetravalent (+4) oxidation states. Furthermore, the analysis reveals that Mo has a particularly high degree of oxidation. The binding energies observed are consistent with the expected zero-valent and oxidation states, confirming that the synthesis process successfully incorporated each of these elements into the mesoporous structure. The compositional ratios obtained by XPS analysis — Pt (13%), Pd (16%), Rh (6%), Ru (3%), Cu (11%), Au (18%), Se (22%), and Mo (11%) — differ slightly from the data obtained by ICP analysis. One reason is that XPS only provides information on the surface composition, whereas ICP measures the overall composition. Additionally, the XPS peaks involve several elements and their different valence states, making the peak deconvolution process complex, which further contributes to the discrepancy (Fig. 4).

Therefore, we used the ICP results to calculate the entropy values. Overall, the above XPS and ICP analyses validate the successful formation of the targeted multi-element mesoporous alloy and provide key insights into the chemical environment of the synthesized material.

Our approach demonstrates that electrochemical deposition facilitates uniform nucleation and grain growth of m-HEAs, making it adaptable to other m-HEA systems. Electrochemical deposition is a process in which metals in an electrolyte (mainly a solution of metal salts) are reduced and deposited onto the surface of a conductive substrate immersed in the solution. This process is driven by an external power source. Specifically, electrons are supplied to the cathode of an electric circuit created by the power source, while metal ions in the solution receive electrons and are reduced, depositing on the surface of the cathode. During this process, a potential slightly lower than the standard electrode potential of the metal ions being reduced is applied to the cathode from the external power source. It is important to note that more than 30 types of metals can be deposited, including those that can be simultaneously deposited as multimetallic alloys.

**Fig. 5:**
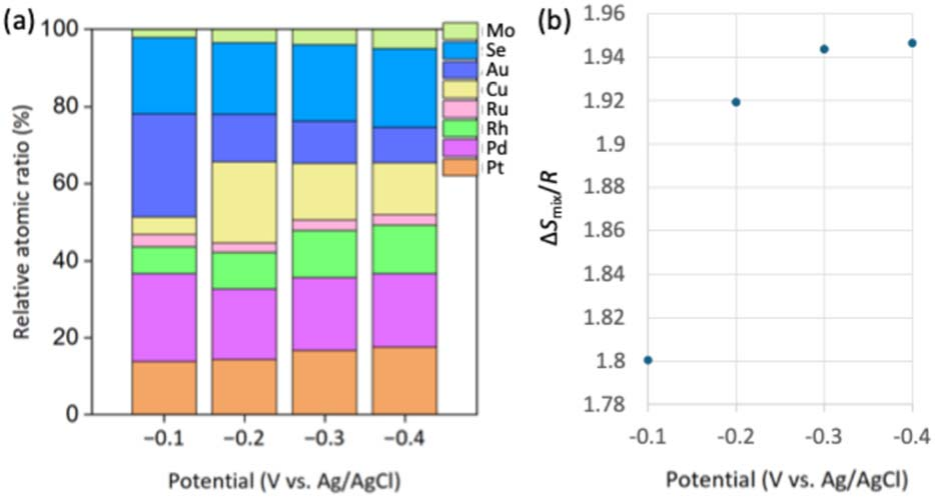
(a) Relative molar compositions of elements (determined by ICP analysis, as shown in Table S3) and (b) corresponding mixed configuration entropy values (Δ*S*_mix_) at different deposition potentials.

The applied potentials play a critical role in the metal electrodeposition process. By changing the electrodeposition potential, significant variations are observed particularly for Pd, Rh, Cu, and Au (Fig. 5a), probably because of the various standard reduction potentials for different elements (Table S3). At −0.1 V (*vs.* Ag/AgCl), Pd and Au were predominantly deposited, with Pd accounting for 22.8% and Au for 26.8%. The mixing entropy at this potential is 1.80*R* (Fig. 5b). When the potential was decreased to −0.2 V (*vs.* Ag/AgCl), Cu deposition increased significantly. While Cu accounted for only 4.5% at −0.1 V (*vs.* Ag/AgCl), it increased to 21.1% at −0.2 V (*vs.* Ag/AgCl). In contrast, the proportion of Au decreased to 12.4%, and Pd also decreased by 5.5%. At this stage, the mixing entropy rose to 1.92*R*. At −0.3 V (*vs.* Ag/AgCl), the Rh content increases to 12.1%, while Cu decreases to 14.7%. In addition, Pt and Se increased by 2.3% and 1.2%, respectively. The mixing entropy further increased to 1.94*R*. At −0.4 V (*vs.* Ag/AgCl), no significant changes were observed, but Pt, Se, and Mo each increased by approximately 1%, while Cu and Au decreased by 1.2% and 1.8%, respectively. By suppressing the excessive deposition of specific metals and maintaining the content of all metals within the range of approximately 3–20%, a high mixing entropy is achieved. Moreover, when electrodeposition is conducted at potentials of −0.2 V (*vs.* Ag/AgCl) or higher, the surface showed fewer cracks and irregularities. However, surface roughness increases significantly at −0.3 V (*vs.* Ag/AgCl) and more negative potentials (Fig. S6). The mixing entropy no longer increases at −0.5 V (*vs.* Ag/AgCl) (Table S4). When the potential exceeds −0.6 V (*vs.* Ag/AgCl), the hydrogen evolution reaction becomes predominant over metal deposition, which adversely affects the deposition process. Therefore, −0.4 V (*vs.* Ag/AgCl) is chosen as the deposition potential in this study.

The electrochemical surface area (ECSA) of the samples was estimated from the electrochemical double-layer capacitance *C*_dl_,^38^ measured by cyclic voltammetry (CV) in the non-faradaic region with varying scan rates from 10 to 120 mV s^−1^ (Fig. S7). The *C*_dl_ was determined from the slope of the capacitive current *versus* scan rate plot. Assuming a specific capacitance of 40 μF cm^−2^ for a flat surface, the ECSA is calculated using the following equation:ECSA = *C*_dl_/0.04 mF cm^−2^ × 0.015 cm^2^

The calculated ECSA of the non-porous sample is 23.55 cm^2^ (*C* = 6.28 mF cm^−2^), whereas the mesoporous sample exhibits a significantly higher ECSA of 39.6 cm^2^ (*C*_dl_ = 10.6 mF cm^−2^). These results indicate a substantial increase in the electrochemically active surface area due to the introduction of mesoporosity. In addition, the interconnected pore network may facilitate rapid mass transport, reducing diffusion limitations for reactants/products, which is essential for high-performance energy conversion/storage devices, such as fuel cells and metal-air batteries, where reaction efficiency hinges on active site availability and species mobility.

## Conclusions

In this study, we have successfully prepared a mesoporous high-entropy alloy film containing eight metals—Pt, Pd, Rh, Ru, Cu, Au, Se, and Mo—for the first time. The alloy exhibits an exceptionally high mixed configuration entropy of 1.95*R*. This approach offers a promising pathway to increase configurational entropy and unlock novel properties in mesoporous multimetallic systems by adjusting the precursor composition. Moving forward, developing materials with even higher configurational entropy by tuning precursor compositions, optimizing deposition conditions, and exploring additional metal species beyond the eight used in this study holds great promise.

## Author contributions

Y. S. and Y. K.: conceptualization, methodology, conducting chemical experiments, and manuscript writing. L. F. and Y. A.: data curation and formal analysis. Y. K. and Y. Y.: supervision, manuscript review, and editing.

## Conflicts of interest

There are no conflicts to declare.

## Supplementary Material

SC-OLF-D5SC04126K-s001

## Data Availability

Supplementary information: all experimental data, including characterization data, synthesis procedures, and supporting figures and tables, are available in the SI. See DOI: https://doi.org/10.1039/d5sc04126k. Additional raw data can be obtained from the corresponding author upon reasonable request.
