# Supplementary material for: Electrochemical deposition of mesoporous high-entropy Pt–Pd–Rh–Ru–Cu–Au–Se–Mo films using polymeric micelle templating
Source: Chem Sci. 2025 Sep 23;16(46):21836–41. doi: 10.1039/d5sc04126k (PMC12533732; doi:10.1039/d5sc04126k)
Supplement: SC-016-D5SC04126K-s001 [file SC-016-D5SC04126K-s001.pdf]

Supplementary Information (SI) for Chemical Science.
This journal is © The Royal Society of Chemistry 2025

Supplementary Information

Electrochemical Deposition of Mesoporous High-Entropy Pt-Pd-Rh-Ru-Cu-Au-Se-Mo Films Using Polymeric Micelle Templating

Yoto Saso, Yunqing Kang*, Lei Fu, Kotaro Yagi, Jungmok You, Yusuke Asakura and Yusuke Yamauchi*

Experimental

Materials. $H_2PtCl_6\cdot 6H_2O$, Na_2PdCl_4, $NaRhCl_6$, $CuSO_4\cdot 5H_2O$, $HAuCl_4\cdot 3H_2O$ and SeO_2 were purchased from Sigma-Aldrich. $RuCl_3\cdot xH_2O$ and $MoCl_5$ were purchased from TCI. Poly (styrene)$_{18,000}$-*b*-Poly (ethylene oxide)$_{7,500}$ (PS_{18000}-*b*-PEO_{7500}) (Note: The numbers in the subscripts give the average molecular weight of the corresponding groups.) were purchased from Polymer Source Inc. Tetrahydrofuran (THF), hydrochloric acid (HCl, 2M) and ethanol (99.5 %) were purchased from Fujifilm Wako Pure Chemical Corporation.

Synthesis of mesoporous HEA films: Mesoporous HEAs films were synthesized by a micelle soft-template method. First, 4 mg of PS_{18000}-*b*-PEO_{7500} was dissolved in 0.6 mL of THF via ultrasonication. Then, 0.5 mL of ethanol, 2.32 mL of deionized water, and 0.08 mL of 2 M HCl were added to form a micelle solution at 40 °C. Subsequently, 0.1 mL each of 40 mM solutions of $H_2PtCl_6\cdot 6H_2O$, Na_2PdCl_4, Na_3RhCl_6, $RuCl_3\cdot xH_2O$, $CuSO_4\cdot 5H_2O$, $HAuCl_4\cdot 3H_2O$, SeO_2, and $MoCl_5$ were sequentially added dropwise into the above solution while stirring in an oil bath heated to 40 °C. The electrodeposition synthesis was carried out at room temperature. The obtained precursor solutions were used as an electrolyte for the typical electrodeposition of mesoporous films at an applied

potential of −0.4 V (vs. Ag/AgCl) for 1200 s. A conventional three-electrode system was employed for electrodeposition, using a Pt wire as the counter electrode and an Ag/AgCl electrode (in 3M KCl) as the reference electrode. Au-Ti-coated Si wafers or carbon paper were selectively used as the working electrode. After electrodeposition, the working electrodes were taken out and immersed in THF for 24 h at room temperature to remove the micelle template. To investigate the effect of deposition potential on the elemental composition and mixing entropy, different electrodeposition potentials were applied (specifically, −0.1, −0.2, and −0.3 V, vs. Ag/AgCl). The same method as described for the typical sample was used, with the only modification being the variation in potential.

Characterizations. SEM observations were conducted using a ZEISS Gemini 560 and a HITACHI S5500 operated at 10 kV. For SEM measurements, the substrate was fixed to the sample stage using carbon tape and observed. SEM-EDS analysis was performed using a Bruker Xflash 6-60 operated at 20 kV. TEM images were obtained using a Talos F200X G2 (Thermo Fisher Scientific) operated at 200 kV. XRD patterns were collected using a SmartLab X-ray diffractometer (Rigaku) over a 2θ range of 30–90°, with a step size of 0.02°, at room temperature using a Cu Kα radiation source (30 kV, 40 mA). XPS spectra were acquired using a PHIQuantes system with an Al Kα X-ray source (1486.6 eV). Binding energy calibration was based on the C 1*s* peak at 284.8 eV. The Shirley background effectively was used for fitting because it can remove irregular and nonlinear background signals, enhancing the useful signal and thereby improving the signal-to-noise ratio and analytical accuracy of the XPS data. The pore-to-pore distance was evaluated using SAXS (Rigaku NANO-Viewer series). Sample compositions were determined by ICP-MS (Agilent 7850).

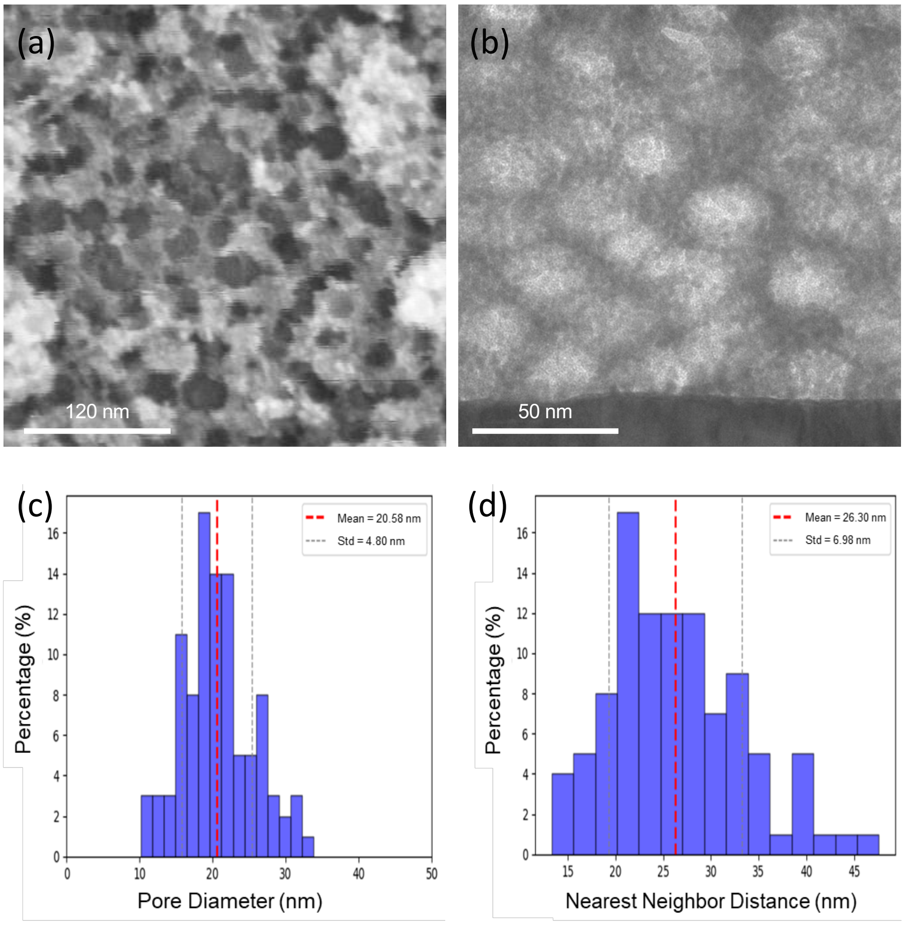

Fig. S1. (a) SEM image, (b) TEM image, (c) pore size distribution, and (d) nearest pore-to-pore distance distribution of m-HEA film.

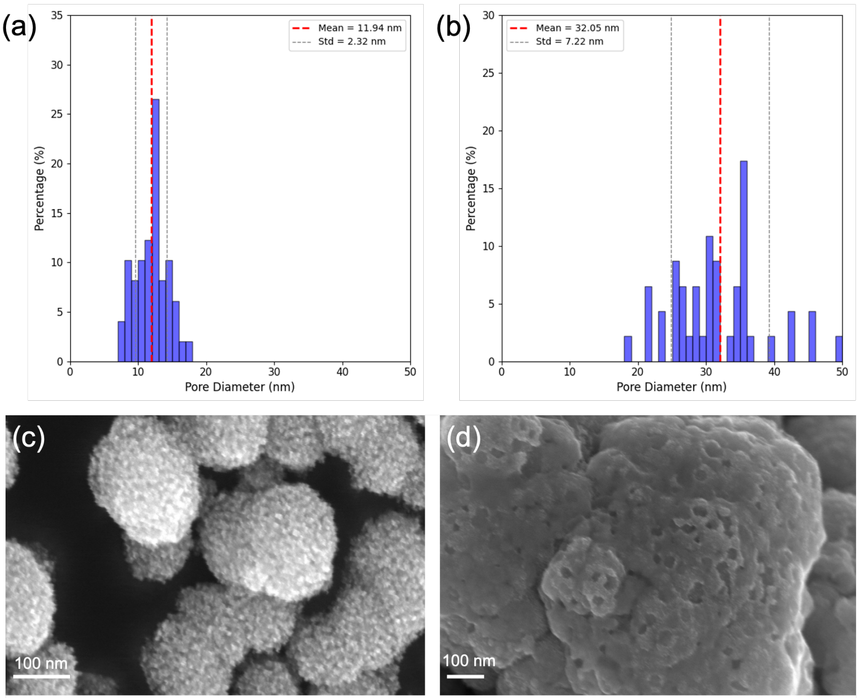

Fig. S2. (a, b) Pore size distributions obtained from SEM images (c) m-HEA film with smaller pores prepared using PS_{3500}-b-PEO_{2500}, and (d) with large pores prepared using PS_{18000}-*b*-PEO_{7500} with the addition of 1, 3, 5-triisopropylbenzene (20 µl).

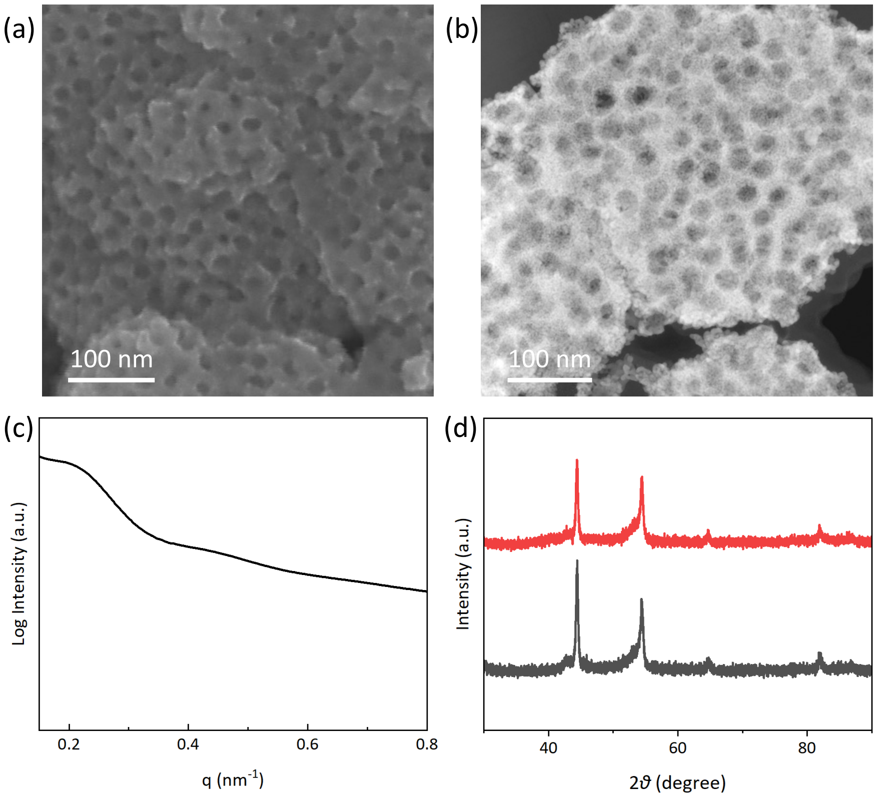

Fig. S3. (a) SEM image, (b) HAADF-STEM, and (c) SAXS pattern of m-HEA film deposited on carbon fiber. (d) XRD patterns of m-HEA film deposited on carbon fiber (red) and pure carbon fiber (black).

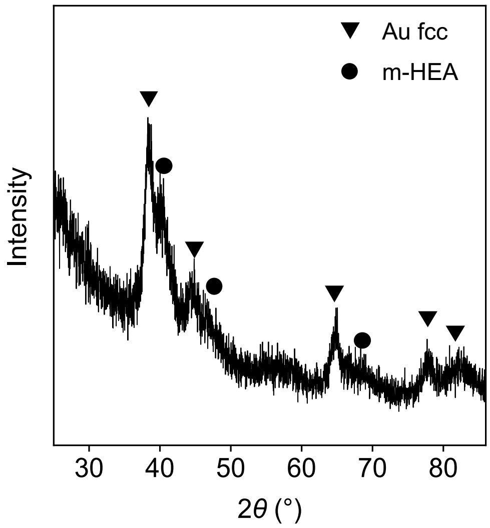

Fig. S4. XRD pattern of m-HEA film deposited on Au-Ti-coated Si substrate.

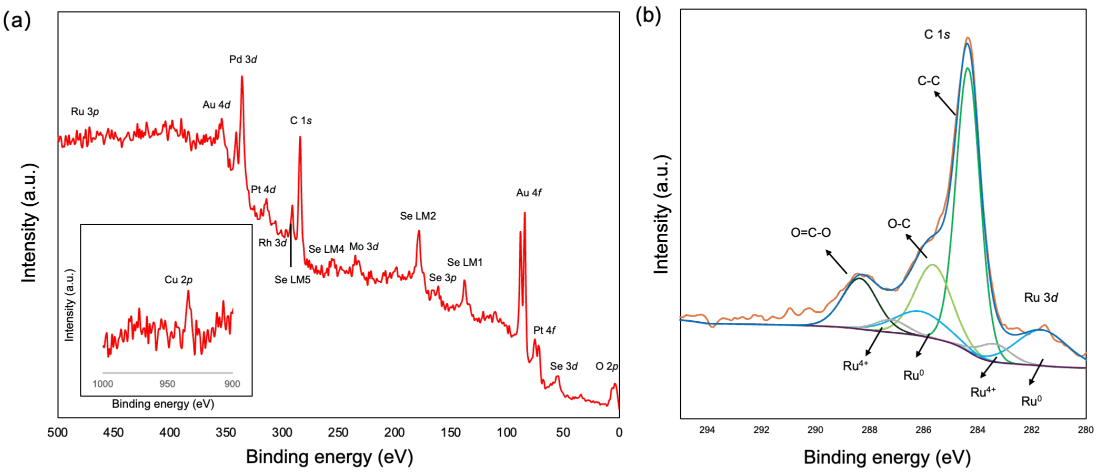

Fig. S5. (a) XPS survey spectrum and (b) high-resolution XPS scan spectra over C 1*s* and Ru 3*d* of the m-HEA film.

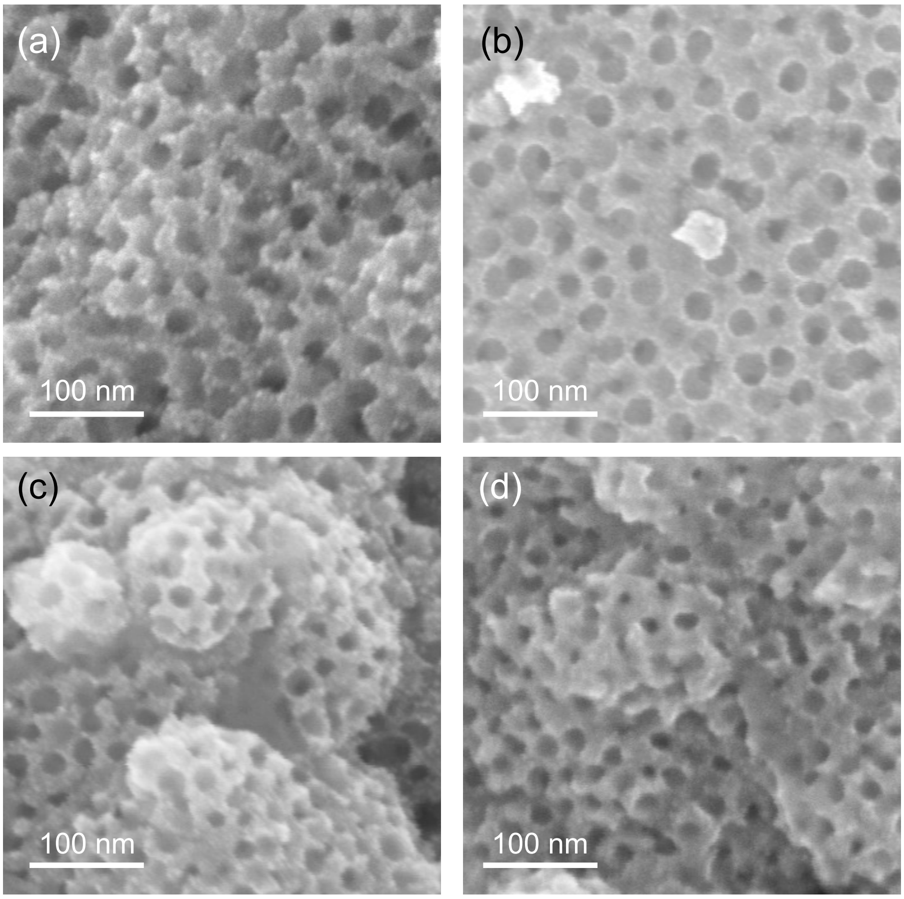

Fig. S6. SEM images of m-HEA film prepared at deposited potential at (a) –0.1 V, (b) –0.2 V, (c) –0.3 V, and (d) –0.4V vs. Ag/AgCl, respectively.

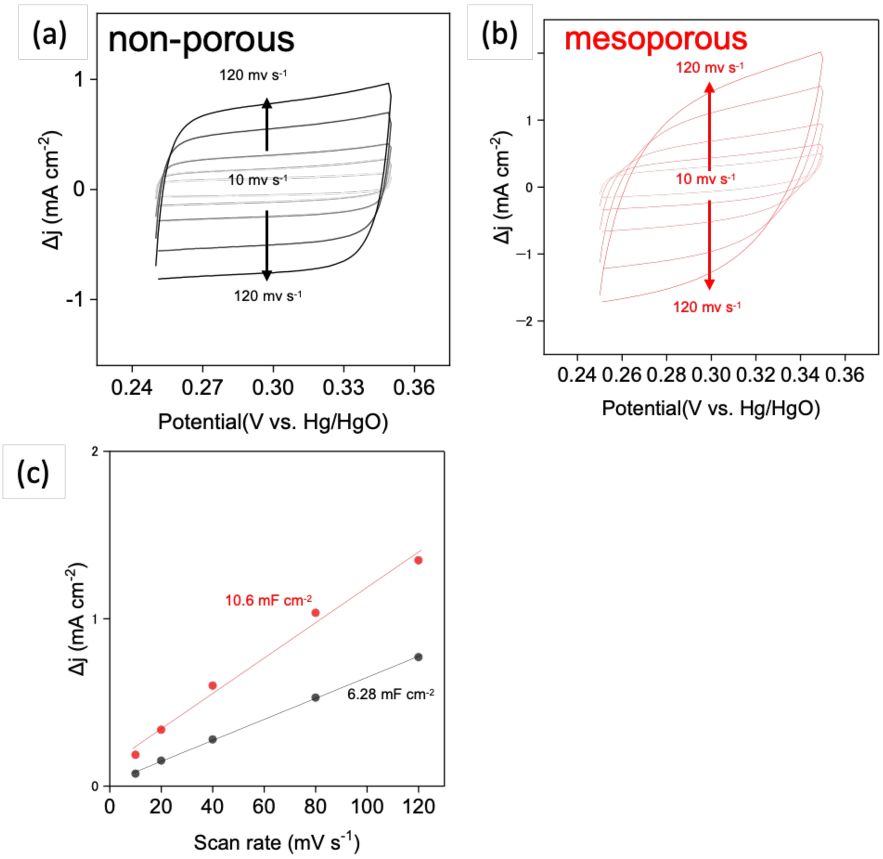

Fig. S7. Cyclic voltammetry measurements in the non-Faradaic region of (a) non-porous HEA and (b) mesoporous HEA (i.e., m-HEA film). (c) The C_{dl} was determined from the slope of the capacitive current versus scan rate plot.

Table S1. The comparation table of the values of mixed configuration entropy of reported mesoporous HEAs.

**Sample's name**	**Elements**	**ΔS_{mix} / *R***	**Method[a]**	**Ref.**
PtPdRhRuCu MMNs	Pt, Pd, Rh, Rh, Cu	1.59	ICP	*Nat. Commun.*, 2023, *14*, 4182.
m-HEA film	Pt, Pd, Rh, Rh, Cu	1.53	ICP	*ACS Nano,* 2024, 18, 27617–27629
HEA10	Pt, Pd, Ru, Mo, Ni	1.50	ICP	*Adv. Sci.*, 2024, 11, 2402518
SCPHEA4	Pt, Pd, Ru, Mo, Ir	1.01	ICP	*J. Am. Chem. Soc.*, 2025, DOI: 10.1021/jacs.5c01260
a-HE-Se	Pt, Pd, Ru, Ru, Ir, Se	1.11	XRF	*Angew. Chem. Int. Ed.*, 2025, 64, e202414786
Porous HEAs	Pt, Pd, Ru, Ru, Ir	1.58	EDS	*ACS Nano*, 2022, 16, 10, 15837–15849
MHEA-PtPdFeCoNi	Pt, Pd, Fe, Co, Ni	1.50	ICP	*Adv. Energy Mater.*, 2024, 14, 2303923
HEA-PdCuAgBiInene	Pd, Cu, Ag, Bi, In	1.38	ICP	*Angew. Chem. Int. Ed.,* 2024, e202410442
PtPdRuIrFeCu mNTs	Pt, Pd, Ru, Ir, Fe, Cu	1.82	ICP	*Chem. Eng. J.,* 2023, 477, 147099
RhAgCuPdPt HEA NPs	Rh, Ag, Cu, Pd, Pt	1.61	XPS	*Nanoscale*, 2025, 17, 6072-6078
FeCoNiMnRu-HCB0.5	Fe, Co, Ni, Mn, Ru	1.60	ICP	*Fuel*, 2025, 391, 134800,
m-HEA film	Pt, Pd, Rh, Ru, Cu, Au, Se, and Mo	1.95	ICP	This work

[a]The ΔS_{mix} value was calculated based on the elemental composition (in at.%) based on the Method. ICP: inductively coupled plasma; XRF: X-ray fluorescence spectroscopy; EDS: Energy Dispersive X-ray Fluorescence; XPS: X-ray photoelectron spectroscopy.

Table S2. The amount of each metal deposited at different voltages obtained from ICP

Element	Applied potential (vs. Ag/AgCl)			
	–0.1 V	–0.2 V	–0.3 V	–0.4 V
Pt	13.9	14.4	16.7	17.5
Pd	22.8	18.3	19.0	19.1
Rh	6.9	9.5	12.1	12.5
Ru	3.2	2.4	2.8	2.9
Cu	4.5	21.0	14.7	13.5
Au	26.8	12.3	11.0	9.2
Se	19.8	18.5	19.7	20.3
Mo	2.1	3.6	4.0	5.0

Table S3. Various reduction potentials for different elements.[a]

Standard Cathode (Reduction) Half-Reaction	Standard reduction potential E° (V vs. SHE)	Reduction potential E (V vs. Ag/AgCl)
$PtCl_4^{2-} + 2e^- \rightleftharpoons Pt(s) + 4Cl^-$	0.73	0.52
$PdCl_4^{2-} + 2e^- \rightleftharpoons Pd(s) + 4Cl^-$	0.53	0.32
$RhCl_6^{3-} + 3e^- \rightleftharpoons Rh(s) + 6Cl^-$	0.44	0.21
$Ru^{3+} + 3e^- \rightleftharpoons Ru\ (s)$	0.60	0.39
$Cu^{2+} + 2e^- \rightleftharpoons Cu\ (s)$	0.34	0.13
$AuCl^{4-} + 3e^- \rightleftharpoons Au\ (s) + 4Cl^-$	1.00	0.79
$H_2SeO_3 + 4H^+ + 4e^- \rightleftharpoons Se(s) + 3H_2O(l)$	0.74	0.53
$Mo^{3+} + 3e^- \rightleftharpoons Mo(s)$	-0.20	-0.41
$MoO_2(s) + 4H^+ + 4e^- \rightleftharpoons Mo(s) + 2H_2O(l)$	-0.15	-0.36
$MoO_4^{2-} + 4H_2O(l) + 6e^- \rightleftharpoons Mo(s) + 8OH^-$	-0.91	-1.12

[a] The standard redox potentials are measured under standard conditions, whereas the actual reduction potentials are influenced by multiple factors, including solvent, temperature, pH, coordination environment, surfactants, and mixing entropy, etc.

Table S4. The compositional ratios and corresponding ΔS_{mix} of m-HEA applied at different potentials obtained from SEM-OES.

Applied potential (V vs. Ag/AgCl)	Pt	Pd	Rh	Ru	Cu	Au[a]	Se	Mo	ΔS_{mix} (R)
**-0.4**	10.2	16.5	11.6	3.0	6.2	23.2	18.8	10.5	1.948
**-0.5**	11.8	15.2	11.8	3.0	5.5	24.4	18.9	9.4	1.937

[a] The Au content was overestimated due to contribution from the Au–Si substrate.
